# Lateral Calcaneal Artery Adipofascial Flap for Reconstruction of the Posterior Heel of the Foot

**DOI:** 10.4055/cios.2009.1.1.1

**Published:** 2009-02-06

**Authors:** Moon Sang Chung, Goo Hyun Baek, Hyun Sik Gong, Seung Hwan Rhee, Won Seok Oh, Min Bum Kim, Kyung Hag Lee, Tae Woo Kim, Young Ho Lee

**Affiliations:** Department of Orthopedic Surgery, Seoul National University College of Medicine, Seoul, Korea.

**Keywords:** Foot, Posterior heel, Soft tissue defect, Lateral calcaneal artery adipofascial flap

## Abstract

**Background:**

Soft tissue defects of the posterior heel of the foot present difficult reconstructive problems. This paper reports the authors' early experience of five patients treated with a lateral calcaneal artery adipofascial flap.

**Methods:**

Between 2003 and 2007, five patients (3 males and 2 females) with soft-tissue defects over the posterior heel underwent a reconstruction using a lateral calcaneal artery adipofascial flap and a full-thickness skin graft. The flap sizes ranged from 3.5 × 2.5 cm to 5.5 × 4.0 cm.

**Results:**

All five flaps survived completely with no subsequent breakdown of the grafted skin, even after regularly wearing normal shoes. The adipofascial flap donor sites were closed primarily in all patients.

**Conclusions:**

Lateral calcaneal artery adipofascial flaps should be included in the surgical armamentarium to cover difficult wounds of the posterior heel of the foot. These flaps do not require the sacrifice of a major artery to the leg or foot, they are relatively thin with minimal morbidity at the donor site, and leave a simple linear scar over the lateral aspect of the foot.

Soft tissue defects of the posterior heel of the foot present difficult reconstructive problems due to the bony prominence, limited availability of local tissue, requirement for specialized tissue, and the limitations imposed by donor site morbidity. Moreover, there are many occasions where primary closure is impossible, even for small defects.1) Moreover, skin grafts must heal, even in cases with exposed bone or tendons and when the recipient sites are stimulated continuously by footwear. In addition, local rotation, advancement, and transposition flaps are limited by the availability of mobile skin. Several types of reverse flow island flaps have been developed in the form of fasciocutaneous or cutaneous flaps but they require sacrifice of an important leg artery and create obvious contour deformities at the donor site. The use of free flaps has improved the ability to cover soft tissue defects. However, the flap bulk, the need for secondary procedures, and the risk of vascular failure are considerable obstacles.

A lateral calcaneal artery skin flap is an axial pattern flap that includes the lateral calcaneal artery, lesser saphenous vein and the sural nerve.2) Since its development in 1981, this flap has been demonstrated to be both an effective and reliable local flap for reconstructing soft tissue defects about the posterior heel and both malle-oli.^3^,4) Modifications of this flap include island arterial flaps,^3^-6) distally based flaps6) and free flaps,7) all of which have a wide variety of clinical applications. Lin et al.8) modified this flap as an adipofascial flap and used it to reconstruct soft tissue defects of the posterior heel as well as the lateral malleolar and lateral supramalleolar areas. This study reports our early experience of five patients treated with this flap for a posterior heel reconstruction.

## METHODS

### Materials

Between March 2003 and April 2007, five patients (3 males and 2 females) with soft-tissue defects over the posterior heel underwent a reconstruction using a lateral calcaneal artery adipofascial flap and a full-thickness skin graft. The soft-tissue defects were caused by acute trauma in three patients and a chronic ulcer in two. The flaps ranged in size from 3.5 × 2.5 cm to 5.5 × 4.0 cm. The patients' ages ranged from 4 to 72 years (mean, 37.2 years), and the follow-up period ranged from 6 to 18 months (mean, 11 months).

### Operative Procedure

Before surgery, the peroneal and lateral calcaneal artery presence and patency were confirmed. The tissue used for the adipofascial flaps was in the same areas as the lateral calcaneal artery skin flap.2) The flap sizes required to cover the heel defects were marked on the skin. Initially, a zigzag skin incision was marked in the central portion of the proposed flap. Surgery was aided with a tourniquet and surgical loupes (× 2.5). The incision was deepened through the skin down to the subcutaneous tissue and superficial venous plexus. The overlying skin was dissected at this venous plane and the lesser saphenous vein and its contributors were identified and preserved on the flap surface of the flap.

After the skin over the proposed flap had been dissected completely, the initial incision for elevation of the fascial side of the flap was made at the posterior aspect of the lateral malleolus and extended proximally along the fibula and lateral tendon of the peroneus longus muscle. Through traction and elevation of the medial edge of the adipofascial flap, the lateral calcaneal vessels and accompanying sural nerve were identified in the suprafascial layer. This neurovascular pedicle was protected and kept in the flap, and used as an anatomical landmark of the proper plane to undermine the fascia. The sural nerve was dissected by incising the fascia and preserved. However, its branches to the flap were separated interfascicularly and retained in the flap. The posterior edge of the flap was incised close to the Achilles tendon near the calcaneus and carried down to the periosteum. Undermining of the fascial side of the flap was straightforward when the dissection was performed immediately over the periosteum of the calcaneus and the dissection could be accomplished through this site.8)

The pivot point of the flap lies proximal to the superior edge of the calcaneus where the lateral calcaneal artery emerges. The proximal base of the flap was not narrowed and included the lateral calcaneal vessels, the superficial venous system and fascicular branches of the sural nerve.8)

**Fig. 1:**
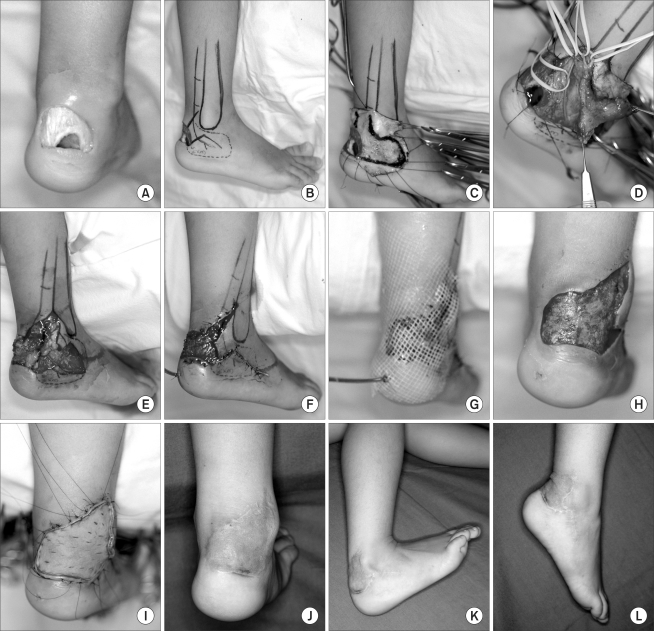
(A) Post-traumatic soft tissue defect with exposure of the calcaneus and Achilles tendon in a 4-year-old female. (B) Design of the lateral calcaneal artery flap showing its vascular supply from the lateral calcaneal artery. The adipofascial flap was obtained from the same territory as the lateral calcaneal artery skin flap. (C) The overlying skin was dissected at this venous plane. (D) The flap was dissected and the lateral calcaneal artery and the lesser saphenous vein could then be visualized. (E) The flap pivot point lies proximal to the superior edge of the calcaneus where the lateral calcaneal artery emerges. (F) The donor site was closed primarily with the preserved skin without grafting. (G) The flap was rotated to the recipient area, and a suction drain was inserted. (H) Appearance of an early granulating bed area at 7 days after surgery. (I) A sheet of full-thickness skin was grafted onto the raw surface of the flap. (J) Reconstructed area showing a good contour and appearance at 12 months postoperatively. (K) Primary healing and linear scar at the donor site at 18 months postoperatively. No functional impairment of ankle motion was evident. (L) The donor site healed primarily without a hypertrophic scar. Note the excellent ankle joint motion.

After rotating the flap to the recipient area, the donor site was closed primarily with preserved skin, and multiple small silastic drains or a suction drain was inserted. A moist, loose 1% framycetin sulfate-impregnated sterile tulle gauze dressing with a wet dressing was simply placed over this flap until the skin graft was performed. On postoperative days 5 to 7, the raw surface of the flap on the recipient site was covered with a full- thickness skin graft (Fig. 1).

## RESULTS

Table 1 shows the patient data. All five flaps had good perfusion and survived completely. No venous congestion was noted but flap edema lasted for 3 days. The skin grafts on the adipofascial flap had taken well in 4 patients. One case (case 3) suffered partial loss of the full-thickness skin graft on the flap that was associated with a thin hematoma beneath the skin graft. However, this healed spontaneously without the need for a secondary graft. In all patients, the donor sites of the adipofascial flap were closed primarily without any functional impairment and healed. One case (case 4) was complicated by a hematoma at the donor site, which was emptied by suction and compression. Marginal desquamation of the donor site wound occurred in one case (case 2) but it subsequently healed. All patients became ambulatory after wound healing, and ankle motion was not restricted. There was no subsequent breakdown of the grafted skin with the regular wearing of shoes.

## DISCUSSION

Adipofascial flaps have inherent shortcomings that warrant consideration.9) These include flap thinness, bleeding or hematoma, monitoring difficulties and skin graft associated problems.^10^-12)

The thinness of adipofascial flaps makes them vulnerable to pressure. Meland and Weimar11) reported that the avoidance of external pressure is essential for maintaining flap viability. They used a protective, clear plastic dome over the flap area, and no dressing material was placed in direct contact with the flaps. In order to avoid this problem, Walton et al.12) dressed the flaps with a thin sheet of petrolatum-impregnated gauze but left them otherwise exposed. We also dressed the flaps using this method but used a 1% framycetin sulphate impregnated sterile tulle gauze instead.

An axial pattern adipofascial flap has a rich blood supply for the vessels to run and form a redundant vascular network within the fascia.10) Therefore, the potential difficulties associated with intraoperative and postoperative bleeding are a valid concern.11) Intraoperative bleeding can be minimized by the careful use of bipolar cauterization. The problems associated a postoperative hematoma beneath flaps is best addressed using small-caliber suction drains, as recommended by Brent and Byrd,13) rather by applying external pressure.11) We currently use a number of small Silastic or suction drains.

Another problem associated with adipofascial flaps is the tendency towards incomplete take-up of the overlying skin graft. Meland and Weimar11) reported that delaying skin grafting for 3 to 5 days allows an early granulating bed to develop on the surface of the adipose tissue, which eliminates the problem of immediate, postoperative oozing beneath the skin graft. In all our cases, the raw surface of the flap on the recipient site was covered with a full thickness skin graft 5 to 7 days after surgery. The main shortcoming of this procedure is that an adipofascial flap or fascial flap must act as an additional skin graft. Most surgeons use a split-thickness skin graft.^10^-^12^,^14^,15) However, Lai et al.16) used an adipofascial turnover flap to reconstruct the dorsum of the foot, and Lin et al.^8^,17) used a full-thickness skin graft obtained from the groin or inguinal areas as a distally based posterior tibial arterial adipofascial flap and lateral calcaneal artery adipofascial flap, which closed primarily. A full-thickness skin graft rather than a split-thickness graft minimizes the breakdown of grafted skin. Full-thickness skin grafts are applied to raw adipofascial flap surfaces to reconstruct the foot and allow the regular wearing of shoes. In this study, a full-thickness skin graft was used in all of the five cases described.

Overall, lateral calcaneal artery adipofascial flaps should be included in the surgical armamentarium to cover difficult wounds of the posterior heel of the foot. They do not require sacrifice of a major artery to the leg or foot, are relatively thin with minimal morbidity at the donor sites, and leave only a simple linear scar over the lateral aspect of the foot. In addition, the flap dissecting technique is straightforward, and has the advantages of better aesthetic results at the donor sites and sural nerve preservation compared with a lateral calcaneal artery skin flap.

Lateral calcaneal artery adipofascial flaps are limited in size but can fill defects of the posterior heel. Moreover, their softness and pliability make them suitable for filling the subcutaneous spaces formed by debridement. In the five patients described in this report, lateral calcaneal artery adipofascial flaps were used to effectively cure intractable defects with minimal donor site morbidity.

## Figures and Tables

**Table 1:**
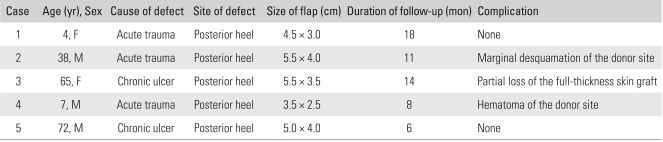
Clinical Data of the Patients

## References

[1] Lee ET (2004). A new wound closure achieving and maintaining device using serial tightening of loop suture and its clinical applications in 15 consecutive patients for up to 102 days. Ann Plast Surg.

[2] Grabb WC, Argenta LC (1981). The lateral calcaneal artery skin flap(the lateral calcaneal artery, lesser saphenous vein, and sural nerve skin flap). Plast Reconstr Surg.

[3] Holmes J, Rayner CR (1984). Lateral calcaneal artery island flaps. Br J Plast Surg.

[4] Yanai A, Park S, Iwao T, Nakamura N (1985). Reconstruction of a skin defect of the posterior heel by a lateral calcaneal flap. Plast Reconstr Surg.

[5] Gang RK (1987). Reconstruction of soft-tissue defect of the posterior heel with a lateral calcaneal artery island flap. Plast Reconstr Surg.

[6] Ishikawa K, Isshiki N, Hoshino K, Mori C (1990). Distally based lateral calcaneal flap. Ann Plast Surg.

[7] Ishikawa K, Kyutoku S, Takeuchi E (1993). Free lateral calcaneal flap. Ann Plast Surg.

[8] Lin SD, Lai CS, Chiu YT, Lin TM (1996). The lateral calcaneal artery adipofascial flap. Br J Plast Surg.

[9] Lee YH, Rah SK, Choi SJ, Chung MS, Baek GH (2004). Distally based lateral supramalleolar adipofascial flap for reconstruction of the dorsum of the foot and ankle. Plast Reconstr Surg.

[10] Jin YT, Cao HP, Chang TS (1989). Clinical application of the free scapular fascial flap. Ann Plast Surg.

[11] Meland NB, Weimar R (1991). Microsurgical reconstruction: experience with free fascia flaps. Ann Plast Surg.

[12] Walton RL, Matory WE, Petry JJ (1985). The posterior calf fascial free flap. Plast Reconstr Surg.

[13] Brent B, Byrd HS (1983). Secondary ear reconstruction with cartilage grafts covered by axial, random, and free flaps of temporoparietal fascia. Plast Reconstr Surg.

[14] Gibstein LA, Abramson DL, Sampson CE, Pribaz JJ (1996). Musculofascial flaps based on the dorsalis pedis vascular pedicle for coverage of the foot and ankle. Ann Plast Surg.

[15] Ismail TI (1990). The dorsalis pedis myofascial flap. Plast Reconstr Surg.

[16] Lai CS, Lin SD, Yang CC, Chou CK (1991). Adipofascial turnover flap for reconstruction of the dorsum of the foot. Br J Plast Surg.

[17] Lin SD, Lai CS, Chou CK, Tsai CW (1992). The distally based posterior tibial arterial adipofascial flap. Br J Plast Surg.

